# Pathology and Cause of Death in Stranded Kogiids: A Retrospective Study from the Canary Islands (1999–2018)

**DOI:** 10.3390/ani16040594

**Published:** 2026-02-13

**Authors:** Pablo Díaz-Santana, Antonio Fernández, Josué Díaz-Delgado, Cristian Suárez-Santana, Óscar Quesada-Canales, Vidal Martin, Eva Sierra, Nakita Câmara, Manuel Arbelo

**Affiliations:** 1 Unit of Veterinary Histology and Pathology, University Institute of Animal Health and Food Safety (IUSA), Veterinary School, University of Las Palmas de Gran Canaria (ULPGC), 35413 Las Palmas de Gran Canaria, Canary Islands, Spain; pablo.diasantana@gmail.com (P.D.-S.); cristian.suarez@ulpgc.es (C.S.-S.); oscar.quesada@ulpgc.es (Ó.Q.-C.); eva.sierra@ulpgc.es (E.S.); nakita.camara@ulpgc.es (N.C.); manuel.arbelo@ulpgc.es (M.A.); 2 Veterinary Pathology Centre, School of Veterinary Medicine, University of Surrey, Guildford GU2 7AQ, UK; 3 Texas A&M Veterinary Medical Diagnostic Laboratory, College Station, TX 77843, USA; 4 Canary Islands Cetaceans Research Centre, Society for the Study of Cetaceans in the Canary Archipelago (SECAC), 35500 Lanzarote, Canary Islands, Spain; vidal@cetaceos.org

**Keywords:** pygmy sperm whale, dwarf sperm whale, cetacean, pathology, wildlife

## Abstract

The scientific understanding of cetacean pathology has advanced significantly in recent decades. However, data concerning the health status of members of the family Kogiidae remains scarce. This study presents a comprehensive pathological assessment and determination of causes of death in 45 stranded kogiids, comprising 35 *Kogia breviceps* and 10 *K. sima*, along the coasts of the Canary Islands between 1999 and 2018. Causes of death (CD) were classified as natural (30/45; 66.6%) or anthropogenic (11/45; 24.4%), while the cause remained undetermined in four cases (9%). Among natural causes, the most prevalent etiologies included trauma (13/30; 43.3%), infectious (7/30; 23.3%), parasitic (5/30; 16.6%), cardiomyopathy (3/30; 10%), malnutrition (1/30; 3.3%), and fetal distress (1/30; 3.3%). Anthropogenic causes were vessel collisions (7/11; 63.6%), interactions with fishing activities (2/11; 18.2%), and foreign body-associated pathology (2/11; 18.2%). Notably, intra- and interspecific traumatic interactions were frequently identified in this cohort, and variable degrees of gross and histologic cardiomyopathic changes were observed in 68.9% of individuals (31/45) without evident sex bias. Septicemia caused by *Clostridium perfringens* and *C. tertium* was confirmed in three cases. Additionally, cervical gill slit adenitis by *Crassicauda* sp. (15/45; 33%) and parasitic gastritis by *Anisakis* sp. (27/45; 60%) were frequent findings. These findings provide novel insights into the pathologic spectrum affecting kogiids and enhance the current understanding of their health status, with implications for diagnostic protocols, stranding response, and conservation strategies.

## Simple Summary

Pygmy and dwarf sperm whales are deep-diving cetacean species that are rarely seen alive. Therefore, most knowledge about their health comes from stranded animals. In this study, we examined 45 stranded whales from the Canary Islands over a 20-year period to elucidate the causes of death and associated diseases. Most deaths were attributed to natural causes, especially injuries caused by interactions with other animals, infections, parasites, and heart disease. Many whales exhibited long-standing cardiac lesions, reiterating heart disease as a common health problem in these species. Parasitic infections of the stomach and the cervical-gill gland were also frequent. Human activities were responsible for a considerable number of deaths, mainly from ship strikes, fishing gear interactions, and ingestion of plastic debris. Various infections and pathologic findings identified in this study had not been previously reported in these whales. Overall, these findings improve understanding of the health problems affecting these little-known whales and highlight the importance of post-mortem examinations to support research informing conservation and protection strategies.

## 1. Introduction

The kogiid whales (family Kogiidae) are medium-sized odontocete cetaceans, including two species: the pygmy sperm whale (*Kogia breviceps*) and the dwarf sperm whale (*K. sima*) [1,2]. Both species are currently classified as least concern (LC) on the International Union for Conservation of Nature (IUCN) Red List, although population trends remain unknown [3,4]. These species have been allocated in the Pacific, Atlantic, and Indian Ocean waters, but there is insufficient information to define the population status [3,4]. Primary dietary sources are deepwater cephalopods (e.g., *Histioteuthidae* and *Cranchiidae* families) with feed depths down to 1200 m for *K. breviceps*, while *K. sima* are thought to feed in shallower water [5,6,7].

Concerning anatomic singularities, both species possess a distinctive colonic diverticulum, or “ink sac”, filled with brownish–red pigment which is released under stressful or life-threatening situations [3,4]. The pygmy sperm whale has a cervical-lateral exocrine gland (“false gill split”) that is commonly parasitized by *Crassicauda magna* [8,9,10]. Specific anatomic investigations using advanced imaging techniques have described unique features, e.g., supracranial basin, spermaceti chamber, hyoid apparatus [11]. *Kogia* spp. are often heavily parasitized by intestinal nematodes such as *Anisakis* spp. and *Terranova cetecola*, as well as larval cestodes of *Clistobothrium delphini* encysted in the blubber [3]. Heavy larval cestode infestations may correlate with shark predation prevalence [5]. Although the overall level of predation on *Kogia* spp. remains unknown, there is evidence of attacks by lampreys, great white sharks (*Carcharodon carcharias*), and killer whales (*Orcinus orca*), with remains of pygmy sperm whales found in killer whale stomachs [5].

Long-term pathology-based health monitoring of uncommon cetacean species is rare. The Kogiidae are among the least studied odontocete taxa [12], largely due to their elusive and deep-diving behavior with short surface intervals, resulting in most biological and pathologic knowledge being derived from stranded individuals [8,12,13,14,15,16,17,18,19,20]. Despite steadily increasing anatomic and diagnostic investigations, longitudinal health assessments remain limited, emphasizing the need for studies focused on their pathology and conservation efforts.

Cetaceans are exposed to environmental stressors, either anthropogenic (e.g., chemical and acoustic pollution, fisheries, maritime traffic, tourism industry) and non-anthropogenic, hereafter ‘natural’ (e.g., biotoxins, pathogens such as bacteria, fungi, parasites, and viruses) [21]. In general terms, physiologic and pathologic knowledge in kogiid whales is limited and fragmentary. Notably, the pygmy sperm whale is possibly the mammal with the highest prevalence of naturally occurring dilated cardiomyopathy. The cause(s) for such prevalence remain uncertain, although derangements in selenium and metallothionein have been argued [19,20,22]. Here, we assessed retrospectively the epidemiology and pathologic findings and causes of death of *K. breviceps* and *K. sima* stranded in the Canary Islands between 1999 and 2018.

## 2. Materials and Methods

### 2.1. Stranding Epidemiologic Data and Necropsy Examination

The required permission for the management of stranded cetaceans anywhere within the Canarian archipelago was issued by the General Subdirectorate for Terrestrial and Marine Biodiversity, Ministry for the Ecological Transition and the Demographic Challenge, Government of Spain. No experiments were performed on live animals because our work was based on dead stranded cetaceans.

Necropsies were conducted on kogiids stranded dead or alive along the coasts of the Canary Islands from 1999 to 2018. Life history data (i.e., age category, sex, body condition [BC], and decomposition condition category [DCC], morphometrics, and stranding conditions of the individuals) were recorded systematically following established criteria [13,14,23] (Table A1). Species identification was based on external morphological features and skeletal anatomical characteristics described for *K. breviceps* and *K. sima* (body size, rostrum shape, dorsal fin morphology, and mandibular features) [3,10,18]. Genetic analysis was not performed. Three age categories based on total body length (TBL) and gonadal macroscopic and histologic appearance were considered: neonate/calf, juvenile, and adult [23,24]. The BC of each animal was established morphologically based on anatomical parameters such as the osseous prominence of the spinous and transverse vertebral processes and ribs, the mass of the epaxial musculature (*longissimus dorsi*, *multifidus*, *spinalis*), and the amount of subcutaneous and cavitary fat deposits, taking account of the species and the age of the animal. These parameters allowed us to classify their BC as good, moderate, poor, and emaciated. The DCC was classified as extremely fresh (individuals known to live-strand and necropsied immediately), fresh, moderate decomposition, advanced decomposition, and very advanced decomposition with mummification or skeletal remains [23,24]. No genetic analysis was performed to distinguish between species, and recognition was eminently based on external and skeletal anatomical features

A standardized necropsy protocol was used [23,24]. Special attention was paid to the cervical gland to establish the presence/absence of *Crassicauda* sp. adult or larval forms. Grading of *Crassicauda* parasitic burden in the cervical gill gland was based on adult and larval nematode counts, wherein <10 individuals represented grade +, 11–20 grade ++, and >21 grade +++.

### 2.2. Statistical Analysis

The association between sex and age category among stranded individuals was assessed using the Fisher–Freeman–Halton exact test in Microsoft Excel (Microsoft Corporation, Redmond, WA, USA) with the Real Statistics add-in. Sex and age category were treated as categorical variables. Statistical significance was set at *p* < 0.05. Generative artificial intelligence (ChatGPT 5.2, OpenAI, San Francisco, CA, USA) was used to assist with the statistical analysis and interpretation of data.

### 2.3. Histopathology and Pathologic Classification

Selected samples collected during necropsy were fixed in 10% neutral buffered formalin, routinely processed, paraffin-embedded, sectioned at 5 μm, and stained with Hematoxylin and Eosin (H&E) for histopathological examination. Additional histochemical techniques, including periodic acid–Schiff (PAS), Masson’s trichrome, and Gram, were performed as needed.

Morphologic diagnoses were established following international and standardized protocols. The prevalence of gross and histologic pathologic findings was assessed per organ/system (Tables S1 and S2). When possible, and in order of relevance if numerous, etiologic diagnoses were also determined. Finally, macroscopic and microscopic post-mortem examination information, together with the stranding circumstances, were scrutinized to determine the “cause of death” (CD) and associated etiologic diagnoses (Table A2). The CD was classified as “natural”, “anthropogenic”, and “not determined”. Major etiologic diagnoses within the natural CDs included: trauma, infectious, parasitic, cardiomyopathy (CMP), and fetal distress. Three major etiologic diagnoses were considered within the anthropic-related CDs: vessel collision (VC), interaction with fishing activities (IFA), and foreign body-associated pathology (FBAP) [13,14,25]. Furthermore, gross and microscopic lesions typically associated with the “stress response syndrome” or “alarm reaction” [26] and “capture myopathy” [27,28,29] were also considered.

### 2.4. Microbiology

Fresh tissue samples (skin, skeletal muscle, lung, prescapular, pulmonary, mediastinal and mesenteric lymph nodes, liver, intestine, kidney, spleen, brain) collected routinely during necropsy, were frozen (−80 °C) and selectively submitted for bacteriologic analysis. These included routine culture and surface plating on routine media, e.g., Columbia blood agar, and preliminary identification of isolates via API^®^ system (API^®^ 20E, API^®^ Rapid 20E, API^®^ Staph, API^®^ 20 Strep, API^®^ Coryne, API^®^ 20A; bioMérieux, Marcy-l’Étoile, France). The tissues cultured and the respective results are recorded in Table S3.

PCR analyses for the detection of cetacean morbillivirus (CeMV) and cetacean herpesvirus (CeHV) followed published protocols [30,31,32,33]. DNA and RNA were simultaneously extracted from 300 µL homogenized tissue samples using pressure filtration with the QuickGene Mini80 system (Kurabo Industries Ltd., Osaka, Japan) and the DNA Tissue Kit S (Kurabo Industries Ltd., Osaka, Japan), with RNA carrier added during the lysis step (Thermo Fisher Scientific, Waltham, MA, USA). When suspected, CeMV was assessed using one-step RT-PCR targeting a 426 bp fragment of the P gene, nested RT-PCR also targeting the phosphoprotein (P) gene, and semiquantitative (sq) RT-PCR targeting a 192 bp region of the Fusion (F) gene. Similarly, CeHV DNA was investigated, when relevant, using nested PCR targeting the DNA polymerase gene. For *Brucella* spp. detection, a duplex sqPCR targeting the IS711 insertion sequence and conventional PCR amplifying a 223 bp fragment of the bcsp31 gene were used [31,34].

### 2.5. Osmium Tetroxide (OsO_4_) and Chromic Acid (H_2_CrO_4_)

Detection of lipid emboli in lung tissue was undertaken using two established histochemical techniques following published methodologies [35]. Available and selected lung samples were fixed in 2% osmium or, alternatively, fixed in chromic acid before paraffin embedding, as both approaches enable preservation and visualization of intravascular neutral lipid droplets and are routinely used in cetaceans to support the diagnosis of ante-mortem blunt trauma associated with vessel collision.

## 3. Results

### 3.1. Stranding Epidemiology

A total of 67 kogiids (*K. breviceps*, *n* = 51; *K. sima*, *n* = 13; unknown kogiid species, *n* = 3) stranded between 1 January 1999 and 31 December 2018. Post-mortem examinations were conducted on 45/67 (67%) animals encompassing 35 *K. breviceps* and 10 *K. sima*. Sex distribution of animals studied was 24 (53.3%) males, 18 (40%) females, and 3 (7%) undetermined. Age categories were as follows: 27/45 (60%) adult, 11/45 (24.4%) juvenile, 7/45 (15.5%) neonate/calf. The Fisher–Freeman–Halton exact test indicated no significant association between sex and age class (exact *p* > 0.05), suggesting these variables were independent in this cohort. Annual strandings through this 20-year period averaged 4 kogiids without a clear yearly stranding bias. Necropsies were performed on 2/45 (4%) extremely fresh, 7/45 (15%) fresh, 18/45 (40%) moderate decomposed, 5/45 (11%) advanced decomposition carcasses, and 13/45 (29%) very advanced decomposition carcasses (mummified or skeletal remains). Five of 45 (11%) animals were stranded alive; 40/45 (88.9%) were stranded dead or were retrieved adrift. Distribution based on BC included 9/45 (20%) good, 12/45 (26.7%) moderate, 9/45 (20%) poor, and 3/45 (6.7%) cachectic animals. The BC could not be determined in 12/45 (26.7%) animals due to advanced decomposition.

The most probable CD was identified in 41/45 (91%) of the examined individuals. The CD was not determined in 4/45 (9%) individuals. Etiologic diagnoses within the natural CDs included trauma (13/30; 43.3%), infectious (7/30; 23.3), parasitic (5/30; 16.6%), CMP (3/30; 10%), malnutrition (1/30; 3.3%), and fetal distress (1/30; 3.3%) while trauma, encompassing vessel collision (7/11; 18.2%) and IFA (2/11; 18.2%), and FBAP (2/11) were the most prevalent within the anthropic CDs. Non-anthropogenic CDs accounted for 30/45 (66.6%) of deaths, whereas anthropogenic activities included 11/45 (24.4%) cases.

### 3.2. Natural Causes of Death

Natural CD was determined in a total of 30 animals, including 27 *K. breviceps* and three *K. sima* (animals no. 2, 3, 5, 8, 10, 11, 12, 13, 14, 16, 17, 22, 23, 24, 25, 26, 28, 29, 31, 32, 33, 34, 35, 36, 37, 38, 39, 42, 44, 45).

**Figure 1:**
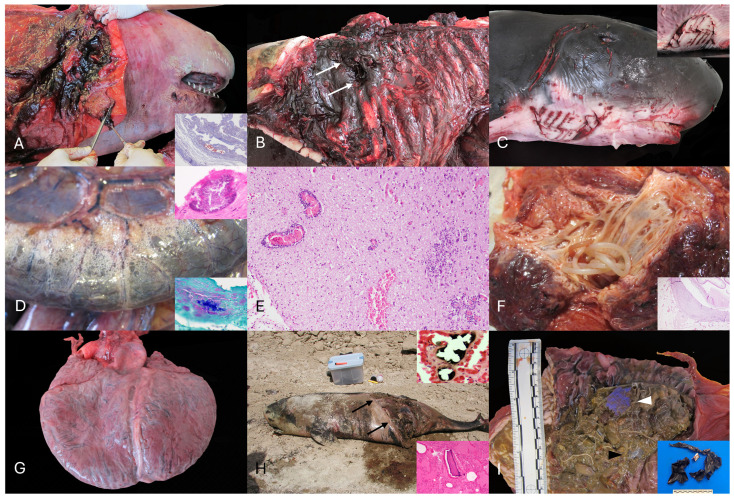
(**A**) Animal no. 28. Intra- and interspecific traumatic interaction. Extensive subcutaneous cervical circumferential hemorrhage extending deep into muscle planes. Inset: Animal no. 27: Intraspecific interaction with killer whale. Fat emboli (Oil Red O). A focal pulmonary subpleural vessel is partially obliterated by a fat embolus (red). (**B**) Animal no. 34: Intra- and interspecific traumatic interaction. Focal perforation with peripheral hemorrhages within the left dorso-lateral aspect of the thoracic cavity (white arrows) and associated lung perforation (non-visible). (**C**) Animal no. 33. Intraspecific traumatic interaction with a shark. Inset: Multifocal cutaneous intraspecific rake marks with dermal hemorrhages and often displaying clean and sharp edges and half-moon disposition (left and ventro-lateral cervical region). (**D**) Animal no. 32. *Clostridium perfringens* infection. The serosa of the small intestine and the mesenterium are multifocally expanded by gas (emphysema; *Pneumatosis intestinalis*). Upper inset, H&E: Fibrinosuppurative peritonitis within the visceral diaphragmatic surface with focal intralesional bacterial aggregate. Lower inset, Gram-stain: Note the Gram-positive intralesional bacteria. (**E**) Animal no. 31. Infectious encephalitis, unknown etiology. Lymphoplasmacytic encephalitis with multiple perivascular cuffing formation (upper left), multiple areas of gliosis, and hemorrhage. (**F**) Animal no. 32. Cervical gill adenitis. The cervical gland is expanded by numerous long nematodes (*Crassicauda* sp.). Inset, H&E: Detail of the pyogranulomatous panniculitis with intralesional transverse section of *Crassicadua* sp. displaying a light eosinophilic cuticle, hypodermis, pseudocoelom, coelomyarian–polymyarian musculature, digestive system lined by cuboidal to columnar cells, and paired uteri (non-visible). (**G**) Animal no. 35. Dilated cardiomyopathy. The heart exhibits a globular shape with marked dilation of the right ventricular chamber and thin right free-ventricular wall (non-visible). Note doble apex. (**H**) Animal no. 7. Vessel collision. Animal at necropsy spot. The abdomen exhibits a longitudinal and oblique section with clear edges and partial evisceration (black arrows). Upper inset, Animal no. 19. Osmium tetraoxide (OsO_4_). Detail of a pulmonary osmiophilic fat embolus within the pulmonary vasculature. Lower inset, Animal 18, H&E: Intravascular osseous fragments. (**I**) Animal no. 30. Foreign body-associated pathology. The keratinized stomach is filled with multiple transparent (black arrowhead) and blue (white arrowhead) plastic materials embedded in abundant *Anisakis* sp. Inset, animal no. 41: Display of gastric foreign plastic material.

Traumatic intra-to-interespecific interaction was identified as the CD in 11 *K. breviceps* and two *K. sima* (animals no. 11, 14, 16, 24, 28, 33, 34, 37, 38, 39, 42, 44, 45). From the adults, a total of five animals were female and five were male. Among females, three were pregnant (animal no. 10 and 11) or exhibited uterine post-partum regression (animal no. 44). Additionally, most of the traumatic events were recorded in animals stranded along the coasts of the western islands (7/13; Tenerife = 4, La Gomera = 2, La Palma = 1). Main macroscopic lesions in these animals were: bone fractures (i.e., skull, mandibular, teeth, vertebral, hyoid apparatus), multiorgan (e.g., pulmonary, central nervous system, lymph nodes) hemorrhage, hemoabdomen, hemothorax, hemopericardium, cutaneous erosions/lacerations, and interspecific rake marks (by killer whales and/or sharks) with occasional extensive loss of muscle bundles (flank, head, thoracic, and central region), hemarthrosis, pulmonary perforation, and pulmonary edema (Figure 1A–C). Parasitic gastritis (e.g., *Anisakis* spp., *P. gasterophilus*) was identified in 10/13 animals, being moderate to severe in 7/13. Most relevant microscopic findings encompassed: multiorgan hemorrhage, more prominently affecting the lungs and central nervous system (CNS), myodegeneration and necrosis of skeletal muscle, and pulmonary edema. Myocardial degeneration and necrosis were common findings in most individuals. Animal no. 14 exhibited pulmonary, cardiac, and lymphatic intravascular gas or fat emboli. Moderate to abundant partially digested ingesta was observed in 7/13 individuals.

Singular cases in this category included an ulcerative dermatitis with vasculitis, epithelial necrosis, with occasional amphophilic intranuclear inclusions identified as herpesvirus through PCR (animal no. 33) and lymphoplasmacytic balanitis with renal urolithiasis (animal no. 34).

Gross and histologic findings aligning with an infectious disease process were identified in five *K. breviceps* and two *K. sima* (animals no. 3, 12, 23, 26, 31, 32, 3). Animal no. 32 exhibited multifocal intestinal serosal emphysema (*pneumatosis intestinalis*), lymphohistiocytic hepatitis with necrosis, splenic necrosis, and multinodal (hepatic, tracheobronchial, retromandibular) necrosuppurative lymphadenitis with intralesional sporulated Gram-positive bacilli (Figure 1D). Animal no. 36 had fibrinosuppurative peritonitis with intralesional Gram-positive, sporulated bacilli. Bacteriologic analysis identified *C. perfringens* in both cases. Animal no. 31 displayed a lymphoplasmacytic meningoencephalitis with myelitis (Figure 1E). Other relevant cases included a fibrinosuppurative omphalophlebitis and peritonitis in animal no. 26, lymphoplasmacytic periportal hepatitis, endometritis, bronchointerstitial pneumonia, and nephritis in animal no. 12, and suppurative bronchopneumonia with type II pneumocyte hyperplasia in animal no. 23. Intravascular pulmonary fat emboli were identified in animal no. 12 by using osmium tetroxide (OsO_4_) staining.

Fatal parasitism was identified in three *K. breviceps* and two *K. sima* (animals no. 13, 10, 17, 22, 25). Suppurative cervical adenitis of the gill slit gland by *Crassicauda* spp. Nematodes, with or without multisystemic nematode egg emboli with regional deep and superficial myositis and fasciitis was observed in animals no. 1, 13, 14, 16, 17, 19, 22, 25, 8, 28, 31, 32, 34, 36, 42 (Figure 1F). From these, animal no. 13 had a 15 cm in diameter abscess of the cervical gill slit. Severe ulcerative gastritis with numerous *Anisakis* spp. was identified in animals no. 17 and 22. A pyogranulomatous cholangiohepatitis with intralesional Brachycladiidae trematodes was also identified in animal no. 17. Additionally, neuroparenchymal vacuolation, including presumptive intramyelinic edema with axonal degeneration, astrocytosis, and neuronal satellitosis were seen within the thalamus and cerebral cortex of this animal. Animal no. 13 exhibited multiple sarcoplasmic masses within skeletal myofibers, associated with changes in sarcolemmal nuclear number and localization, variation in myofiber size and shape, and altered fiber type proportions. Animal no. 25 exhibited moderate catarrhal bronchitis with intraluminal nematodes (undetermined species). A hepatocellular carcinoma was noted in this animal.

Severe CMP with subsequent multiorgan failure was identified as the main cause of death in three *K. breviceps* (animals no. 2, 5, 35). Two were adults (male and female), and one was a juvenile male. Prominent gross changes included dilation of the right ventricle (Figure 1G) and pulmonary congestion, edema, and hemorrhage, along with chronic passive hepatic congestion. Frequent histologic features comprised: acute cardiomyocyte degeneration, contraction band necrosis, myocardial fiber disarray, juxtanuclear vacuolization, cardiomyocyte hypertrophy, and subendocardial and myocardial fibrosis. Additional findings in these animals included: cerebral perivascular edema (animal no. 2), hepatic periportal fibrosis with ductular reaction (animal no. 5), and hydrothorax (animal no. 2). A ventricular septal defect was present in animal no. 35. Overall, 31 animals (68.9%) displayed gross and/or histologic features consistent with CMP (Table S4). Of these, 15 were males, 14 females, and two of undetermined sex. Most affected individuals were adults (*n* = 19), followed by juveniles (*n* = 6), and calves (*n* = 6). Acute cardiomyocyte degeneration and/or contraction band necrosis were identified in 20 cases (44%), and cardiomegaly was observed in 14 individuals (31%), including nine males, four females, and one animal of undetermined sex; among these, nine were adults, four juveniles, and one calf. Pulmonary edema (26/45; 57.8%) and hepatic congestion (18/45; 40%) were common in this group, particularly those with cardiomegaly, in which pulmonary edema was present in almost all cases.

Animal no. 8 exhibited marked emaciation with diffuse skeletal muscle atrophy and nearly complete lack of ingesta. Mild multiorgan parasitism, including suppurative cervical lymphadenitis, and the presence of an early-term fetus were also documented.

Fetal distress was diagnosed in animal no. 29, a newborn with typical findings of neonatal development, e.g., folded fins and fetal folds, open umbilicus, non- erupted teeth, and non-ossified cranial sutures. Histologically, pulmonary edema and fetal atelectasis, and moderate, acute myodegeneration and necrosis were seen in the skeletal and diaphragmatic muscles.

### 3.3. Anthropogenic Pathologic Categories

VC involved 7/45 (%) *K. breviceps* (animals no. 7, 9, 15, 18, 19, 27, 43). Main gross findings in these animals, usually severe, included: sharp and blunt trauma, polyostotic fractures, more prominently in the cephalocervical region (e.g., skull, mandible, vertebrae; animals no. 7, 9, 18, 19, 27, 43) (Figure 1H), cutaneous lacerations (animals no. 9, 10, 19), sectioning or amputation (animals no. 15, 27 and 43), pulmonary hemorrhage (animals no. 9, 10, 18, 19, 27, 43), cavitary and multiorgan hemorrhage (animals no. 18 and 27), and evisceration (animal no. 27). Microscopically, pulmonary and CNS hemorrhage, contraction band necrosis, acute flocculent, granular, or discoid skeletal myodegeneration were common. Animal no. 19 exhibited multiorgan intravascular gas emboli, while chromic acid (H_2_CrO_4_) demonstrated intravascular fat emboli in animal no. 27. Gastrointestinal parasitism by *Anisakis* spp. and/or *Pholeter gastrophilus* was identified in animals no. 9 and 19. Dilated cardiomyopathy with fibrosis, hypertrophy, and contraction band necrosis was present in animals no. 19 and 27.

Fatal IFA involved 2/45 animals, including one *K. breviceps* and one *K. sima* (animal no. 1, 4). Trauma was the main etiologic diagnosis in these animals. Major gross findings associated with by-catch included: comminute fracture of the base of the skull and occipital bone with extensive subdural hematoma (animal no. 1) and fractures of the skull, mandible, and tympanic bulla together with cutaneous marks compatible with entanglement (animal no. 4). Microscopically, acute skeletal muscle degeneration and necrosis, pulmonary edema and hemorrhage, and CNS hemorrhage, were described. Animal no. 1 exhibited marked parasitism of the skeletal epaxial and hypaxial muscles by *Crassicauda* spp., hydroureter, and dilated cardiomyopathy.

FBAP was determined in 2/45 *K. breviceps* (animals no. 30 and 41). Macroscopically, the glandular stomach of both animals was filled with moderate, heterogeneous, hard, and firm plastic-based fragments along with ulcerative gastritis and myriads of *Anisakis* spp. (Figure 1I).

## 4. Discussion

This study provides the most comprehensive pathology-based assessment of kogiids from the Atlantic Ocean to date. Epidemiologic data from 67 stranded whales were compiled, and detailed necropsy and histopathologic examinations were performed on 45 individuals (35 *K. breviceps*, 10 *K. sima*). By integrating gross, microscopic, and ancillary laboratory test results, we determined causes of death and characterized the pathogenesis of major natural and anthropogenic conditions. When applicable, traumatic and other lesions were interpreted using veterinary forensic criteria [33]. This approach provides a rigorous framework for evaluating health status, mortality drivers, and species-specific vulnerabilities in these poorly studied species [10,11,13,14,18,20,36,37].

### 4.1. Natural Causes of Death

Among natural CDs, traumatic intra- and interspecific interaction was the most prevalent entity. The pathogenesis of fatal sharp-blunt trauma in cetaceans reflects the general principles of mechanical injury; the sudden application of kinetic energy exceeding tissue tolerance results in acute disruption of soft tissues, vascular rupture, and rapid hemodynamic and respiratory compromise, with high-energy ramming and compression producing severe internal polytrauma even when external lesions are limited [36,38,39,40]. The spatial distribution of these events in the Canary Islands and the absence of a clear sex predilection mirror patterns previously documented for cetaceans stranded in the same region [40]. In these animals, the main post-mortem findings were consistent with sharp and blunt force polytrauma; numerous animals exhibited abundant undigested ingesta and frequent cutaneous rake marks, in agreement with previous studies [40]. Cutaneous tooth rake marks may be the most representative finding in this entity, as the other findings could be found in other categories involving fatal trauma [40]. Predation by killer whales (*Orcinus orca*) was identified based on deep, circular rake marks spaced 2–3 cm apart, and by massive loss of soft and bone tissues in two animals. Killer whales are versatile top predators known to prey upon numerous odontocetes and mysticetes [41,42,43,44,45,46,47] with documented fatal attacks on kogiids in different geographic regions [48,49]. These species are seasonal visitors in the Canary Islands, following tuna migration and frequently interacting with pygmy sperm whales [40,50]. Our findings align with the proposed higher susceptibility of deep-diving cetaceans to killer whale attacks [37].

Other less frequent but relevant findings included hepatocellular intracytoplasmic hyaline globules, reported in acute systemic stress responses [51], hyaline casts and acute pigment-associated tubular injury, documented in life stranding events and compatible with hypoxia, hematuria and/or myoglobinuria [27,28]. Intravascular and parenchymal gas embolism in animal no. 14 was interpreted as compatible with a decompression syndrome secondary to the traumatic event and abrupt alteration of the diving pattern [40,52]. Gas analysis could not be performed in this animal due to logistical impediments [53,54].

Parasitic gastritis by *Anisakis* spp. and *P. gastrophilus* was the most prevalent comorbidity in 60% of evaluated animals, likely contributing to a reduced overall health status and a potentially increased vulnerability to traumatic interactions. Chronic mechanical injury from these parasites promotes ulceration, fibrosis, and protein-losing gastropathy, changes well documented in helminth-infected cetaceans [55,56,57]. Gastric anisakiasis is widely recognized in cetaceans and shows increasing global incidence [55,56,57]. An extensive study on short-beaked common dolphins (*Delphinus delphis*) reported an incidence of ulcerative gastritis with intralesional *Anisakis* spp. in 74% of examined animals, with molecular identification of *Anisakis simplex* and *A. pegreffii* [55]. Similar high morbidity has been described in other cetacean species [56,57]. In the present study, identification of the species or genotype of *Anisakis* involved was not performed.

Animal no. 10 exhibited a fibrinosuppurative peritonitis following rupture of a hepatic abscess. Implication of Brachychladiidae trematodes is presumed, as trematode-associated abscess rupture has been previously reported in odontocetes [13,58,59].

A herpesviral dermatitis was diagnosed in animal no. 33. Cutaneous herpesvirus has been described and characterized in a variety of cetacean species [60,61,62,63,64], but it remains largely uncharacterized in kogiids [62,65,66]. Therefore, this case provides additional evidence of herpesviral infection in this family, contributing to the body of knowledge of this ubiquitous microorganism, underscoring the value of continued monitoring [31,34,67]. A lymphoplasmacytic balanitis was identified in animal no. 34. Although a viral agent is highly suspected, immunohistochemical or molecular techniques are required for final confirmation.

Bacteriologic analysis identified septicemia by *C. perfringens* in animals no. 32 and 36, exhibiting multisystemic lesions. The pathogenesis of *C. perfringens* infection is largely toxin-driven, with rapid proliferation under anaerobic conditions and release of α-toxin, perfringolysin O, and β-toxin (type C), which induce endothelial injury, mucosal necrosis, hemorrhage, and systemic toxemia [68,69]. Clostridial diseases have been rarely documented, causing disease in both captive and free-ranging cetaceans [70,71,72,73,74]. Reported historical lesions in cetaceans included necrotizing myositis, emphysema, and dryness, mainly involving skeletal muscles, serofibrinous exudate, multiorgan hemorrhage, and myocarditis [70,71,72,73,74]. Notably, pneumatosis intestinalis has been associated with gas-producing *C. perfringens* (e.g., type A) in humans and several animal species [75,76,77,78]. Clostridial toxin characterization for strain typification was not performed in these animals. *C. tertium* was isolated from a dwarf sperm whale (animal no. 3). Clostridium tertium has been rarely identified in cetacean species [14,79]. Our findings expand the body of knowledge of clostridial diseases in cetaceans, documenting the first *C. perfringens* infection in pygmy sperm whales and a rare *C. tertium* case in one dwarf sperm whale. A lymphoplasmacytic meningoencephalitis with myelitis was diagnosed in animal no. 31. PCR assessment for CeMV and CeHV yielded negative results. Further evaluations are required to elucidate the underlying etiology. Other cases without confirmed etiology included omphalophlebitis with peritonitis (animal no. 26), hepatitis, endometritis, nephritis, and pneumonia (animal no. 12 and no. 23).

Cervical slit adenitis by *Crassicauda* spp. was identified in most animals. A recent study provided an extensive morphologic and histologic description of the gill slit gland, only described in pygmy sperm whales [9]. The gill slit has been identified as a frequent site for infection by *C. magna* [8,10]. This giant nematode (~3 m-long in adults) reproduces inside this exocrine gland and uses it to release the eggs into the environment [9]. In our cases, gross and histological findings together with body location, align with a moderate to severe infestation by *C. magna*. Such severe parasitism of the gill split can alter social interaction, mating behaviors, or territoriality, given the described functions for this gland [6,7,8]. Crassicaudiasis is a significant parasitic disease of cetaceans with predilection for vascular, renal, subcutaneous, and glandular (e.g., cervical gill slit, mammary gland) tissues, which may exhibit species-specific patterns of tissue tropism and serious health implications [8,80,81,82,83,84,85,86].

Cardiomyopathy was the most probable CD in three *K. breviceps*. In this condition, reduced myocardial function leads to congestive heart failure, with progressively impaired forward flow and venous congestion [87]. Left-sided failure results in pulmonary venous congestion and alveolar edema, while right-sided or global failure causes chronic passive hepatic congestion and systemic congestion [87]. Neurohumoral activation further increases circulating volume and capillary pressures, promoting interstitial and serous effusions [87]. In our study, the recurrent association of cardiomyopathic lesions with pulmonary edema and hepatic congestion is therefore consistent with terminal congestive cardiac failure rather than isolated myocardial injury. Cardiomyopathy is well documented in kogiids, but the etiopathogenic mechanisms remain unknown [19,20,22,88]. Upregulation of metallothioneins and sequestering of transition metals (e.g., selenium) have been described in kogiids with CMP, arguably due to adaptive cardioprotection [89]. Bossart et al. [19] documented gross and histologic features of CMP and myocardial degeneration occurring in approximately 50% and 40%, respectively, of examined kogiids, mainly adult males. In contrast, our cohort showed 31 (69%) individuals with lesions compatible with CMP, without evident sex distribution bias and a broader age range. The high prevalence of chronic cardiac lesions with remodeling, particularly among adult individuals, supports the interpretation of these as hallmarks of pathologic CMP rather than species-specific physiologic adaptations. Further investigations into the etiopathogenic mechanisms of CMP in kogiid whales, including genetic analyses, are warranted.

Animal no. 13 exhibited multiple sarcoplasmic masses within skeletal muscle, resembling lesions described in chronic myopathies of humans and domestic animals [90]. Malnutrition or starvation was the CD in animal no. 8. Prolonged energy deficiency leads to depletion of adipose and muscular reserves, impaired gluconeogenesis, and systemic metabolic stress with elevated pro-inflammatory cytokines, particularly TNF-α, which accelerates proteolysis and lipolysis, compounding tissue catabolism [91]. This animal had profound skeletal muscle atrophy, minimal ingesta, myositis, and fasciitis by *Crassicauda* spp., and cutaneous lesions consistent with intra- and interspecific interactions. Malnutrition in cetaceans is a critical health challenge linked to reduced reproductive capacity, increased susceptibility to comorbidities, and progression to multiorgan dysfunction [13,14,92,93,94,95,96,97]. Poor body condition is described in species undergoing prolonged periods of starvation during migration or breeding seasons [98,99,100]. In this case, early pregnancy likely imposed additional energetic demands that might have aggravated health decline [101].

Fetal distress was the CD in a neonate (animal no. 29). Fetal distress arises from impaired placental or fetal oxygenation, leading to hypoxia, redistribution of fetal blood flow, and increased intestinal peristalsis with meconium passage and aspiration, producing atelectasis, chemical pneumonitis, and metabolic injury [102,103]. Our findings in this case are consistent with previous cases of fetal distress associated with various etiologies in cetaceans [13,29,103,104].

### 4.2. Anthropogenic Causes of Death

Vessel collision is a major threat to cetaceans with a growing incidence driven by increasing maritime traffic and vessel speed [105,106,107]. Certain species, including the fin whale (*Balaenoptera physalus*), humpback whales (*Megaptera novaeangliae*), North Atlantic right whales (*Eubalaena glacialis*), or sperm whales (*Physeter macrocephalus*), are particularly vulnerable [39,105,107,108]. In our cohort, adult males were overrepresented among VC cases, contrasting with previous studies that describe higher susceptibility in pregnant females and neonates in some cetacean populations, providing novel epidemiologic data of ship collision in *K. breviceps* [13,105,107,108,109,110]. VC risk is strongly influenced by boat speeds and species-specific surface behaviors, including resting, foraging, nursing, and socializing on the surface [105,106,108,111,112,113,114,115]. The Atlantic Ocean harbors the highest occurrence of documented ship strikes, with the Canary Islands identified as a major collision hotspot due to heavy maritime traffic and high densities of cetacean populations [13,14,109,113,114]. Gross and histologic findings in our cases are consistent with propeller and hull impacts, leading to catastrophic tissue damage and rapid cardiovascular collapse [36,107,116,117,118]. With occasional pulmonary fat embolism further supporting antemortem trauma, even in decomposed carcasses [34,35,109]. VC emerged as the leading anthropogenic CD in this study, underscoring its significance as a conservation concern for pygmy sperm whales, for collision-related mortality could exceed recruitment and drive population decline, either alone or in synergy with other stressors [39,114].

IFA was the CD in animals no. 1 and 4. Bycatch is the leading anthropogenic threat to cetaceans worldwide, with gillnets, trawls, and longlines responsible for most entanglement-related mortality [25,118,119,120]. Small odontocetes are especially vulnerable, often dying from asphyxia, while larger mysticetes may endure long-term entanglement injuries [25,39,118,120,121,122]. In addition, fishing gear can also cause sharp injuries, including hook or harpoon punctures [121,123]. Underreporting is widespread, especially in gillnet fisheries, where observer-based estimates routinely exceed fisher logbook reports [124,125]. Although adult shallow divers are typically overrepresented in IFA records from the Canary Islands [121], the involvement of two juveniles in our cohort aligns with proposed increased susceptibility linked to behavioral immaturity, nearshore foraging, and longer surface intervals [120,121]. IFAs are rarely documented in kogiids due to their cryptic behavior and identification challenges at sea [20,126,127,128]. These findings contribute to the limited knowledge on the pathologic consequences of fisheries interactions in kogiids and highlight the need to consider such impacts on less-reported species.

FBAP was the cause of death in animals no. 30 and 41. Marine debris ingestion poses a widespread threat to cetaceans, with records in over 63% of species [129,130,131]. Deep-diving species and young age are recognized as risk factors for foreign body ingestion, likely due to limited foraging experience and reduced ability to discriminate prey from acoustically mistaken anthropogenic debris [13,94,132,133]. Plastic ingestion has been previously reported in kogiids [5,134]. In this study, both animals exhibited moderate to abundant firm and hard plastic fragments in the glandular stomach associated with ulcerative gastritis, a common concurrent lesion in FBAP [94,135]. In animal no. 30, numerous *Anisakis* spp. were embedded within the plastic mass, causing gastric obstruction that likely resulted in impaired digestion, malnutrition, and increased susceptibility to comorbidities [13,94,136]. Animal no. 41 had intralesional filamentous bacteria associated with *Splendore–Hoeppli* phenomenon within the gastric ulcer [56]. These findings reinforce early age and deep-diving behavior as major risk factors for foreign-body ingestion in kogiids.

## 5. Conclusions

In conclusion, this study provides a comprehensive pathologic assessment of stranded *Kogia breviceps* and *K. sima* over a two-decade period in the Canary Islands. Natural causes of death accounted for most deaths, with intra- and interspecific trauma representing the most frequent pathologic category, followed by infectious and parasitic disease, cardiomyopathy, malnutrition, and fetal distress. Anthropogenic causes were also relevant and included vessel collisions, fishing interactions, and foreign body-associated pathology. Intra- and interspecific traumatic interactions were overrepresented in this cohort. Cardiomyopathic lesions were highly prevalent (69%) without any evident sex distribution bias. Cervical gill adenitis by *Crassicauda* spp. and gastritis by *Anisakis* sp. were prevalent parasitic diseases. Septicemia caused by *Clostridium perfringens* and *C. tertium* were novel findings for these species. Collectively, these findings expand current knowledge on kogiid causes of death and pathology, provide important biological population-level information, reinforce the critical role of continuous stranding surveillance and post-mortem investigations in conservation planning and evidence-based management strategies, while shedding light on both natural and human-derived threats that may contribute useful information for conservation efforts.

## Data Availability

The data presented in this study are available on request from the corresponding authors.

## Supplementary Materials

The following supporting information can be downloaded at: https://www.mdpi.com/article/10.3390/ani16040594/s1, Table S1. Bacterial isolations in 45 kogiid whales (*Kogia breviceps*, *K. sima*) stranded din the Canary Islands (1999–2018). Table S2. Most prevalent gross findings in 45 kogiid whales (*Kogia breviceps*, *K. sima*) stranded in the Canary Islands (1999–2018). Af: number of affected animals, Ev: number of evaluated animals. Table S3. Most prevalent histologic findings in 45 kogiid whales (*Kogia breviceps*, *K. sima*) stranded din the Canary Islands (1999–2018). Af: number of affected animals, Ev: number of evaluated animals. Table S4. Gross and histologic pathologic findings in cardiac tissue from 45 kogiid whales (*Kogia breviceps*, *K. sima*) stranded din the Canary Islands (1999–2018).

## Appendix A

**Table A1:** Biological and epidemiological data of 45 kogiids examined in this study. Sex: female (F), male (M), not determined (ND). Age: neonate/calf (NC), juvenile (Jv), adult (Ad). Stranding date (SD; mm/dd/yy). Type of stranding (TS; dead: D, alive: A). Stranding location, island (IS): Gran Canaria (GC), Fuerteventura (FT), Lanzarote (LZ), Tenerife (TF), La Gomera (LG), El Hierro (EH), La Palma (LP), La Graciosa (LGra). Decomposition condition categories (DCC): Extremely fresh carcass (EF), fresh (F), moderate decomposition (MD), advanced decomposition (AD), mummified or skeletal remains (MSR).

No	Species	Sex	Age	SD	TS	IS	DCC
1	*K. breviceps*	M	Jv	11/19/99	D	FT	F
2	*K. breviceps*	F	Ad	09/27/00	A	TF	MD
3	*K. sima*	F	Ad	07/10/01	D	GC	MSR
4	*K. sima*	M	Jv	01/15/02	D	GC	MSR
5	*K. breviceps*	M	Ad	07/31/02	D	LGra	MD
6	*K. sima*	M	Jv	05/23/03	D	LG	MD
7	*K. breviceps*	M	Jv	06/30/03	D	TF	MSR
8	*K. breviceps*	F	Ad	09/25/05	D	GC	MD
9	*K. breviceps*	M	Jv	2/19/06	D	GC	MSR
10	*K. breviceps*	M	Ad	3/17/06	D	LZ	MD
11	*K. breviceps*	F	Ad	3/31/06	D	LG	MD
12	*K. sima*	F	Ad	5/23/06	D	FT	F
13	*K. breviceps*	F	Ad	8/13/06	D	FT	AD
14	*K. breviceps*	F	Ad	4/6/07	D	TF	MD
15	*K. breviceps*	ND	Ad	6/20/07	D	GC	MSR
16	*K. breviceps*	F	Ad	8/29/07	D	LZ	MD
17	*K. breviceps*	M	Ad	12/2/07	A	GC	EF
18	*K. breviceps*	F	Jv	1/31/08	D	TF	AD
19	*K. breviceps*	M	Ad	6/27/08	D	LZ	MD
20	*K. sima*	ND	Ad	7/1/10	D	GC	MSR
21	*K. breviceps*	M	NC	7/11/10	D	FT	MSR
22	*K. breviceps*	M	Ad	2/12/11	D	FT	AD
23	*K. breviceps*	M	NC	3/28/11	D	FT	MD
24	*K. breviceps*	M	Ad	1/3/12	D	LG	AD
25	*K. breviceps*	F	Ad	3/26/12	D	LZ	AD
26	*K. breviceps*	F	NC	7/26/12	A	TF	EF
27	*K. breviceps*	ND	Ad	13/04/13	D	TF	MSR
28	*K. breviceps*	M	Ad	29/04/13	D	GC	MD
29	*K. sima*	F	NC	14/08/13	D	GC	MSR
30	*K. breviceps*	F	NC	20/03/14	A	GC	F
31	*K. breviceps*	M	Ad	20/11/14	D	FT	F
32	*K. breviceps*	M	Jv	02/12/14	D	GC	MD
33	*K. breviceps*	F	Jv	09/12/14	D	FT	MD
34	*K. breviceps*	M	Ad	13/06/15	D	GC	MSR
35	*K. breviceps*	M	Jv	14/09/15	A	FT	F
36	*K. breviceps*	M	Ad	26/12/15	D	LZ	MD
37	*K. sima*	F	Ad	20/05/16	D	TF	F
38	*K. breviceps*	M	Ad	15/11/16	D	FT	MD
39	*K. sima*	F	NC	02/12/17	D	TF	MD
40	*K. breviceps*	M	NC	16/01/18	D	FT	MSR
41	*K. breviceps*	F	Jv	29/01/18	D	TF	F
42	*K. breviceps*	M	Jv	24/06/18	D	LP	MD
43	*K. breviceps*	M	Ad	09/08/18	D	FT	MSR
44	*K. sima*	F	Ad	23/08/18	D	LZ	MSR
45	*K. sima*	M	Ad	04/09/18	D	TF	MD

**Table A2:** Pathologic categories and etiologic entities in 45 kogiid whales (*Kogia breviceps*, *K. sima*) stranded din the Canary Islands (1999–2018). Af: number of affected animals, Ev: number of evaluated animals.

Cause of Death	Etiologic Entities	Cases	Af/Ev (%)
*Natural or Non-anthropogenic*			
	Trauma (intra-interspecific)	11, 14, 16, 24, 28, 33, 34, 37, 38, 39, 42, 44, 45	13/45 (27%)
	Infectious	3, 12, 23, 26, 31, 32, 36	7/45 (15%)
	Parasitic	13, 10, 17, 22, 25	4/45 (9%)
	Cardiomyopathy	2, 5, 35	3/45 (7%)
	Malnutrition	8	1/45 (2%)
	Fetal distress	29	1/45 (2%)
*Anthropogenic*			
	Vessel collision	7, 9, 15, 18, 19, 27, 43	8/45 (18%)
	Interaction with fishing activities	1, 4	2/45 (4%)
	Foreign body-associated pathology	30, 41	2/45 (4%)
*Undetermined*		6, 20, 21, 40	4/45 (9%)
