# Supplementary material for: Pathology and Cause of Death in Stranded Kogiids: A Retrospective Study from the Canary Islands (1999–2018)
Source: Animals (Basel). 2026 Feb 13;16(4):594. doi: 10.3390/ani16040594 (PMC12937270; doi:10.3390/ani16040594)
Supplement: Supplementary file 1 [file animals-16-00594-s001.zip › animals-4125308-supplementary.pdf]

Table S1. Bacterial isolations in 45 kogiid whales (*Kogia breviceps, K. sima*) stranded din the Canary Islands (1999-2018).

Animal reference	Specie	Target tissue	Isolated organism
No. 3	*K. sima*	- Kidney, CNS<br>- Liver	- *Clostridium tertium*<br>- *Staphylococcus epidermidis*
No. 24	*K. breviceps*	- Spiracle	- *Mycoplasma* sp.
No. 32	*K. breviceps*	- Liver, Mediastinal lymph node, lung, kidney	- *Cl. perfringens*
No. 36	*K. breviceps*	- Kidney, abdominal fluid, CNS<br>- CNS	- *Cl. Perfringens*<br>- *Cetobacterium ceti*

Table S2. Most prevalent gross findings in 45 kogiid whales (*Kogia breviceps, K. sima*) stranded in the Canary Islands (1999-2018). Af: number of affected animals, Ev: number of evaluated animals

**Gross findings**	**Case No.**	**Af/Ev (%)**
	Integumentary and musculoskeletal systems	
Fracture (skull, mandibular, teeth, vertebral, hyoid)	1, 4, 5, 7, 8, 34, 7, 17, 18, 19, 20, 24, 27, 32, 34, 36, 37, 38, 39, 43, 44	21/45 (46.7%)
Hemorrhage/hematoma	82, 8, 11, 14, 17, 18, 24, 25, 26, 28, 33, 34, 35, 36, 37, 38, 39, 42, 43, 44	20/45 (44.4%)
Erosions/lacerations	1, 2, 6, 11, 12, 13, 16, 19, 26, 31, 33, 35, 38, 41, 5, 45	16/45 (35/6%)
Shark bites (antemortem, postmortem)	3, 8, 13, 14, 15, 16, 20, 24, 33, 34, 40, 42, 44, 45	14/45 (31%)
Intra-interspecific interaction	1, 8, 11, 14, 16, 24, 32, 33, 37, 42, 44, 45	6/45 (13.4%)
Cutaneous linear cuts	2, 8, 11, 12, 14, 34	6/45 (13.4%)
Extensive soft-tissue loss with exposed bone and parallel 2–3 cm marks consistent with killer whale trauma	44, 45	2/45 (2.3%)
Non-ossified mandibular symphysis.	39	1/45 (2.2%)
Early development features (Fetal folds, separated skull sutures, non-fused mandibular symphysis)	29	1/45 (2.2%)
- Endoparasitism		
Clistobothrium delphini (blubber)	1, 5, 11, 12, 13, 14, 16, 17, 19, 22, 23, 24, 25, 28, 32, 34, 35, 36, 38, 42, 43, 44	22/45 (48.9%)
Crassicauda sp. (false gill adenitis, panniculitis, myositis, fasciitis)	1, 13, 14, 16, 17, 19, 22, 25, 8, 28, 31, 32, 34, 36, 42	15/45 (33.3%)
Clistobothrium grimaldi (Blubber)	8, 11, 12, 19, 22, 27, 42	7/45 (15.6%)
- Ectoparasitism		
Penella balaenopterae	1, 2, 19, 28, 30, 33, 42	7/45 (15.6%)
Conchoderma sp.	30	1/45 (2.2%)
Xenobalanus sp.	1	1/45 (2.2%)
	Cardiovascular system	
Cardiomegaly	1, 2, 5, 11, 16, 20, 23, 25, 28, 31, 32, 33, 35, 45	14/45 (31.1%)
Hydropericardium	13, 19, 22, 25, 27, 41	6/45 (13.4%)
Congestion with endocardial hemorrhage	6, 41	2/45 (2.3%)
Hemopericardium	28, 36	2/45 (2.3%)
Persistent ductus arteriosus	26, 41	2/45 (2.3%)

Diffusely pale myocardium with thickened left ventricle	27	1/45 (2.2%)
Respiratory system		
Pulmonary edema (congestion/hemorrhage)	1, 2, 4, 5, 6, 11, 13, 14, 16, 17, 19, 20, 23, 24, 26, 28, 30, 31, 32, 33, 35, 36, 37, 41, 42, 45	26/45(57.8%)
Pulmonary emphysema	6, 9, 10, 20, 21, 22, 24, 28, 41	9/45 (20%)
Hydro- / pyothorax	2, 13, 19, 22, 25, 27, 35, 41	8/45 (17.7%)
Hemothorax serosanguineous effusion	36, 39, 18, 24, 28, 34, 37	7/45 (15.6%)
Pulmonary granulomas/pyogranulomatous pneumonia	8, 13, 16, 17, 24, 25, 32	7/45 (15.5%)
Pulmonary atelectasis	20, 24, 26, 28, 39	5/45 (11.1%)
Lungworms	22, 25	2/45 (2.3%)
Pulmonary abscess	20	1/45 (2.2%)
Pulmonary perforation	34	1/45 (2.2%)
Pleural adherences;	27	1/45 (2.2%)
Nematodes in the spiracle	32	1/45 (2.2%)
Pleuritis	2	1/45 (2.2%)
Alimentary system and peritoneal cavity		
- Keratinized, glandular, pyloric stomachs		
- Ingesta		
- Moderate/abundant partially digested (cephalopods, beaks, otoliths and/or crustacea) (rarely in esophagus: 9, 10)	5, 6, 8, 9, 10, 11, 12, 13, 14, 15, 16, 17, 18, 19, 20, 23, 24, 25, 31	19/45 (42.2%)
- Absent	18, 26, 26, 39	4/45 (6.7%)
- Scarce (squid beaks, remains of crustaceans and fish)	8	1/45 (2.2%)
- Gastric pyrosomids	33	1/45 (2.2%)
- Gastric priapulids	30	1/45 (2.2%)
Anisakidae (gastric chambers, esophagus, pharynx)	1, 5, 11, 12, 13, 14, 16, 17, 19, 20, 22, 23, 24, 25, 30, 31, 32, 33, 34, 35, 36, 37, 38, 41, 42, 43, 45	27/45 (60%)
Gastric (keratinized, glandular, pyloric) ulcers	3, 5, 6, 8, 11, 12, 16, 17, 19, 20, 22, 23, 25, 27, 30, 31, 32, 33, 35, 37, 41, 42, 45	23/45 (51.1%)
Pholeter gastrophilus	12, 22, 24	3/ 45 (6.7%)
Foreign body	30, 41	2/45 (2.3%)
Intestines		
Enteric acantocephalans	28	1/45 (2.2%)
Enteric cestodes	28	1/45 (2.2%)
Liver		
Hepatomegaly/ hepatic congestion	1, 5, 6, 8, 11, 12, 14, 18, 28, 38, 41	11/45 (24.4%)
Brachycladiidae	12, 17, 20	3/ 45 (6.7%)
Hepatic infarct	32	1/45 (2.2%)

Hepatic capsular scarring and congestion	31	1/45 (2.2%)
Presumptive hepatic atrophy (small accessory lobe)	33	1/45 (2.2%)
Gas embolism	6	1/45 (2.2%)
Hepatic lipidosis	20	1/45 (2.2%)
	Peritoneal cavity	
Hemoabdomen	11, 22, 28, 32, 34, 38, 44	7/45 (15.6%)
Ascites	1, 19, 25, 36	4/45 (6.7%)
Peritonitis	14, 36	2/45 (2.3%)
	Lymphoid system	
Lymphadenomegaly	1, 14, 16, 17, 30, 32, 34, 33, 40, 44	10/45 (22.2%)
Lymph node congestion	12, 13, 22	3/ 45 (6.7%)
Ectopic spleens	12, 38	2/45 (2.3%)
Retromandibular and prescapular necrotizing lymphadenitis	32	1/45 (2.2%)
Mesenteric lymph node hemorrhage	32	1/45 (2.2%)
Suppurative mesenteric lymphadenitis	11	1/45 (2.2%)
Splenomegaly	22	1/45 (2.2%)
Necrotizing splenitis	32	1/45 (2.2%)
	Urinary system	
Dilated urinary bladder	1, 2, 5, 6, 8, 33, 37, 38	8/45 (17.7%)
Renal congestion/hemorrhage	1, 14, 5, 6, 8	5/45 (11.1%)
Hydroureter	1, 19	2/45 (2.3%)
Nephrolithiasis	42	1/45 (2.2%)
Ulcerative cystitis	33	1/45 (2.2%)
Serosanguineous fluid in the bladder with floccular content	37	1/45 (2.2%)
	Reproductive system	
- Male		
Prostate: Multifocal cystic dilatations	36	1/45 (2.2%)
Fibrinous balanopositis	43	1/45 (2.2%)
- Female		
Pregnancy	2, 8, 8, 11, 16, 25	5/45 (11.1%)
Regressing (post-partum involution)	12, 44	2/45 (2.3%)
	Nervous system	
Meningeal, spinal cord, and brain congestion/hemorrhage	2, 4, 5, 6, 8, 19, 22, 23, 25, 33, 37, 38, 39, 41, 44	15/45 (33.3%)
Pituitary hemorrhage	19	1/45 (2.2%)
	Sense organs	
Hemorrhages in the mandibular fat	31, 32	2/45 (2.3%)
Hemorrhages of pterygoid sacs and acoustic sac	8, 42	2/45 (2.3%)
Gas bubbles in the anterior chamber (eye);	30	1/45 (2.2%)
Bilateral ocular hemorrhage	14	1/45 (2.2%)
Collapse of the eyeball into the orbital fossa	16	1/45 (2.2%)

Table S3. Most prevalent histologic findings in 45 kogiid whales (*Kogia breviceps, K. sima*) stranded din the Canary Islands (1999-2018). Af: number of affected animals, Ev: number of evaluated animals

Histologic findings	Case No.	Af/Ev (%)
	Integumentary system	
Granulomatous/ pyogranulomatous myositis; fasciitis; panniculitis; adenitis (cervical gill) by *Crassicauda* sp.	17, 19, 24, 30, 32, 36, 38, 42	8/45 (17.8%)
Lymphoplasmacytic dermatitis with one or more of the following findings: epithelial necrosis, vacuolization of keratinocytes, acantholysis, vasculitis, papillary congestion, intranuclear acidophilic to amphophilic inclusions.	26, 30, 31, 34, 35	5/45 (11.1%)
Necrohemorrhagic ulcerative dermatitis (intra-interaction marks)	34, 45	2/45 (2.3%)
Lymphoplasmacytic and eosinophilic dermatitis with intralesional diatom algae	16	1/45 (2.2%)
Neutrophilic panniculitis with thrombosis and hemorrhage; multifocal ulcers with neutrophilic infiltrate and superficial epithelial necrosis.	26	1/45 (2.2%)
	Musculoskeletal system	
Skeletal muscle (*RA/LD*): Acute degeneration (e.g., discoid) and necrosis (e.g., floccular, segmental) with hemorrhages, occasional mineralization, regeneration attempts (nuclear rowing)	11, 12, 13, 14, 16, 17, 19, 20, 22, 24, 25, 26, 27, 28, 29, 30, 31, 32, 33, 34, 35, 36, 37, 38, 39, 40, 43, 44, 45	29/45 (64.4%)
Diaphragm: Acute, mild, multifocal segmental myocyte necrosis with occasional discoid degeneration, regeneration attempts,	20, 22, 24, 25, 29, 30, 31	7/45 (15.6%)
Toraco-lumbar/ cervical/ retromandibular: Moderate, multifocal chronic piogrnaulomatous and necrotizing fascitis/ miositis/ panniculitis with intralesional *Crassicauda* sp.	28, 32	2/45 (2.3%)
Interfibrilar *Sarcocystis* sp.	35	1/45 (2.2%)
Musculature contiguous to occipital fracture: multifocal hemorrhages with segmental necrosis, central nuclear rowing, and perimysial and endomysial edema.	37	1/45 (2.2%)
Focal discoid degeneration.	38	1/45 (2.2%)
Histiocytic myositis	42	1/45 (2.2%)
- *Diaphragm*		
Multifocal segmental necrosis with occasional nuclear rowing	31	1/45 (2.2%)
Fibrinosuppurative and lymphoplasmacytic serositis with intralesional sporulated bacillary bacteria	36	1/45 (2.2%)

Cardiovascular system		
- *Heart*		
Acute cardiomyocyte degeneration; necrosis (contraction band necrosis)	12, 13, 16, 17, 19, 20, 22, 23, 24, 25, 26, 27, 30, 35, 37, 40, 41, 42, 44, 45	20/45 (44.4%)
Congestion; hemorrhage	5, 12, 14, 17, 22, 30, 31, 37, 38, 39, 41, 45	12/45 (26.7%)
Cardiomyocyte hypertrophy	11, 12, 14, 16, 19, 20, 42	7/45 (15.6%)
Endocardial, myocardial fibrosis	5, 24, 28, 39, 42, 43	6/45 (13,4%)
Juxtanuclear vacuolization	14, 16, 25, 28, 37, 42	6/45 (13.3%)
Intercellular edema	16, 17, 23, 30, 31	5/45 (11.1%)
Intravascular bacteria	16, 36, 37	3/ 45 (6.7%)
Intravascular dilatations (gas-fat)	14	1/45 (2.2%)
Minimal neutrophilic myocarditis	42	1/45 (2.2%)
Anitschkov-like cells (regeneration)	45	1/45 (2.2%)
Respiratory system		
- *Lung*		
Edema (alveolar, perivascular, pleural)	6, 11, 13, 14, 16, 18, 5, 33, 37, 32, 29, 23, 20, 45, 41, 35	16/45 (35.6%)
Hemorrhage	39, 13, 14, 16, 19, 34, 37, 28, 41, 44, 45, 35, 2, 5, 11	15/45 (33.3%)
Multifocal bronchial/olar mucosal/ cartilage mineralization.	2, 5, 11, 12, 15, 17, 24, 25, 13, 14, 16, 19, 20, 22	14/45 (31.1%)
Atelectasis	2, 3, 17, 6, 13, 35, 32, 28, 29, 27, 43, 31	12/45 (26.7%)
Lymphoplasmacytic interstitial pneumonia/bronchitis with epithelial necrosis and bronchial submucosal oedema	17, 19, 20, 28, 31, 32, 41	7/45 (15.6%)
Gas, fat embolism (OsO4, H_2CrO_4 +)	12, 14, 17, 19, 27	5/45 (11.1%)
Emphysema	3, 13, 21, 28, 31	5/45 (11.1%)
Interstitial and pleural pulmonary fibrosis	6¸13, 14	3/ 45 (6.7%)
Interstitial and pleural pulmonary fibrosis	6¸13, 14	3/ 45 (6.7%)
Hyaline membranes	31, 45	2/45 (2.3%)
Granulomatous pneumonia; intralesional nematode eggs	20, 38	2/45 (2.3%)
Bacteria (alveoli, bronchi)	16, 19	2/45 (2.3%)
Osseous emboli	18	1/45 (2.2%)
Suppurative bronchopneumonia with type II pneumocyte hyperplasia	23	1/45 (2.2%)
Keratin spicules of keratin	26	1/45 (2.2%)
Villous pleural fibrosis	12	1/45 (2.2%)
Alimentary system		
- *Tongue*		
Hemorrhage	31, 34	2/45 (2.3%)
Focal mineralized granuloma with fibrosis, atrophy	39	1/45 (2.2%)

Interstitial fibrosis, myocyte atrophy and occasional regeneration	20	1/45 (2.2%)
Lymphoplasmacytic and histiocytic glossitis	33	1/45 (2.2%)
Exophytic hyperkeratosis, keratin pearls, amphophilic intranuclear	34	1/45 (2.2%)
- *Esophagus*		
Focal ulcerative lymphoplasmacytic and hemorrhagic esophagitis with intralesional bacillary bacteria	41	1/45 (2.2%)
Sarcocystis sp.	35	1/45 (2.2%)
histiocytic and neutrophilic oesophagitis	35	1/45 (2.2%)
Locally extensive submucosal hemorrhage	39	1/45 (2.2%)
- *Liver*		
Hepatic congestion (marked panlobular sinusoidal dilatation, atrophy of hepatic cords, loss of hepatocytes, hepatocellular dissociation)	5, 6, 8, 12, 13, 17, 19, 22, 23, 26, 28, 30, 31, 32, 37, 41, 42, 44	18/45 (40%)
Hepatic lipidosis (steatosis, micro and macrovacuolar)	1, 2, 5, 11, 12, 13, 26, 30, 33, 35, 37, 38, 41, 42, 44, 45	16/45 (35.6%)
Intracytoplasmic hyaline hepatocellular globules	5, 6, 12, 13, 17, 19, 22, 26, 31, 32, 35, 37, 38, 42	14/45 (31.1%)
Liver (intraparenchymal) hemorrhage	18, 14, 16, 17, 28, 30, 37, 38, 42, 44, 45	11/45 (24.4%)
Hemosiderosis	13, 16, 17, 31, 32, 38, 45	7/45 (15.6%)
Lymphoplasmacytic/ eosinophilic cholangiohepatitis with fibrosis and bile duct hyperplasia	5, 12, 13, 17, 35	5/45 (11.1%)
Intracanalicular/intravascular (eggs) trematodes	13, 17, 20, 38	4/45 (8.9%)
Multifocal necrotizing lymphohistiocytic hepatitis	32, 38	2/45 (2.3%)
Intravascular nematode eggs (*Crassicauda* sp.)	8	1/45 (2.2%)
Multifocal-coalescing sinusoidal mid-zonal regions hemorrhage	33	1/45 (2.2%)
Bile duct hyperplasia with occasional cholestasis	33	1/45 (2.2%)
Hepatocellular necrosis	19	1/45 (2.2%)
- *Keratinized stomach*		
Ulcerative gastritis	20, 24, 27, 30, 31, 42, 45	7/45 (15.6%)
Ulcerative gastritis with intralesional bacteria	19, 38, 41	3/ 45 (6.7%)
Lymphoplasmacytic gastritis	17, 30	2/45 (2.3%)
- *Glandular stomach*		
Granulomatous/ lymphoplasmacytic gastritis with intralesional nematodes	17, 20, 22, 24, 32, 35, 37, 38, 41	9/45 (20%)
Pyogranulomatous gastritis by *P. gastrophilus*	12, 16, 19, 20, 25, 30, 40	7/45 (15.6%)
With intralesional algae	25	1/45 (2.2%)
Intravascular (lymphatic) nematode	17	1/45 (2.2%)
Lymphoplasmacytic serositis	22	1/45 (2.2%)
Ulcerative gastritis with foreign body	30	1/45 (2.2%)

Lymphoplasmacytic gastritis	31	1/45 (2.2%)
Necrosuppurative gastritis with intracytoplasmic amphophilic inclusion in parietal cells	33	1/45 (2.2%)
Necrosuppurative gastritis with *Splendore-Hoeppli* bacteria	41	1/45 (2.2%)
- *Pyloric stomach*		
Pyogranulomatous gastritis by *P. gastrophilus*	12, 19, 20, 28, 30	5/45 (11.1%)
Lymphoplasmacytic gastritis with intralesional nematode/ nematode eggs	17, 45	2/45 (2.3%)
Mucosal mineralization	22	1/45 (2.2%)
Necrosuppurative gastritis with vasculitis	33	1/45 (2.2%)
Submucosal hemorrhage with nematode eggs	42	1/45 (2.2%)
- *Intestines*		
Lymphoplasmacytic and histiocytic enteritis	12, 13, 16, 17, 20, 31	6/45 (13.3%)
Fibrionsuppurative serositis with abundant intralesional sporulated bacillary bacteria	12, 36	2/45 (2.3%)
	Lymphoid system	
- *Spleen*		
Extramedullary hematopoiesis	12, 16, 26, 44	4/45 (8.9%)
Splenic hemorrhage	11, 12, 14, 32	4/45 (8.9%)
Hemosiderosis, hemosiderophages	13, 38, 42	3/ 45 (6.7%)
Lymphoid depletion	26, 38, 42	3/ 45 (6.7%)
Sinus histiocytosis	16, 26	2/45 (2.3%)
Bacterial intravascular emboli	16	1/45 (2.2%)
Arteriosclerosis	24	1/45 (2.2%)
Necrotizing splenitis with lymphoplasmacytic serositis	32	1/45 (2.2%)
- *Lymph nodes (Preescapular, mediastinal, pulmonary, mesenteric, retromandibular, hepatic)*		
Centrofolicular hyalinosis	5, 17, 19, 22. 28, 32, 35, 38	8/45 (17.8%)
Hemosiderosis	11, 12, 14, 16, 20, 33	6/45 (13.3%)
Hemorrhage	16, 32, 33, 34, 35, 38	6/45 (13.3%)
Blood drainage	5, 12, 17, 19, 22	5/45 (11.1%)
Intravascular/ sinusal nematode eggs	14, 16, 17, 28, 32	5/45 (11.1%)
Gas-Fat dilations	11, 14, 17, 20, 41	5/45 (11.1%)
Erythrophagocytosis	16, 17, 26, 33	4/45 (8.9%)
Lymphoid hyperplasia	35, 38, 41	3/ 45 (6.7%)
Lymphoid depletion	5, 19, 26	3/ 45 (6.7%)
Lymphocytolysis	26, 33, 41	3/ 45 (6.7%)
Granulomatous lymphadenitis with intralesional ciliated protozoa	19	1/45 (2.2%)
Histiocytic and necrosuppurative lymphadenitis with sporulated bacillary bacteria	32	1/45 (2.2%)
	Urinary system	

- *Kidney*		
Congestion, hemorrhages	5, 6, 7, 11, 13, 16, 17, 22, 26, 28, 32, 33, 35, 36, 37, 38, 41, 45	18/45 (40&)
Medullary tubular mineralization	6, 13, 17, 24, 25, 28, 32, 37, 38	9/45 (20%)
Glomerular, tubular proteinosis, casts	13, 17, 28, 33, 36, 38, 42, 43	8/45 (17.8%)
Pigmentary tubulonephrosis	27, 28, 33, 38	4/45 (8.9%)
Glomerular, tubular dilatation	16, 17, 32, 38	4/45 (8.9%)
Interstitial fibrosis	13, 28, 42, 45	4/45 (8.9%)
Glomerulosclerosis	12, 13, 36	3/ 45 (6.7%)
Crassicauda sp. eggs	16, 17	2/45 (2.3%)
Lymphoplasmacytic interstitial nephritis	12, 32	2/45 (2.3%)
Pelvic nephrolithiasis (with urothelial hyperplasia)	42	1/45 (2.2%)
Membranous glomerulonephritis	5	1/45 (2.2%)
Intravascular bacteria	16	1/45 (2.2%)
Interstitial bacillary sporulated bacteria	36	1/45 (2.2%)
- *Urinary bladder*		
Congestion, hemorrhage	23, 32, 33	3/ 45 (6.7%)
Edema	26	1/45 (2.2%)
Incomplete paravesical (umbilical) arterial sclerosis	26	1/45 (2.2%)
Lymphoplasmacytic cystitis	33	1/45 (2.2%)
Epithelial necrosis with cytoplasmic inclusion bodies	30	1/45 (2.2%)
Reproductive organs (Male/ Female)		
- *Penis*		
Lymphoplasmacytic balanitis, hyperplasia, vacuolization with/without inclusions and chromatin shifting	32, 34	2/45 (2.3%)
- *Prostate*		
Lymphoplasmacytic interstitial prostatitis with fibrosis	36	1/45 (2.2%)
Vascular hyalinosis	36	1/45 (2.2%)
Mineralizations	36	1/45 (2.2%)
- *Ovary*		
Congestion, hemorrhage	37, 39	2/45 (2.3%)
- *Uterus*		
Congestion, hemorrhage	12, 37	2/45 (2.3%)
Lymphoplasmacytic endometritis with luminal bacteria	12	1/45 (2.2%)
Arteriosclerosis with multifocal medial arterial mineralization	13	1/45 (2.2%)
Endometrial hyperplasia with minimal neutrophils	44	1/45 (2.2%)
- *Vagina*		
Submucosal hemorrhages	33	1/45 (2.2%)
Central nervous system		

- *Brain*		
Parenchymal and perivascular hemorrhage	17, 22, 23, 24, 26, 33, 35, 37, 38, 41, 42, 44, 13, 14, 16	15/45 (33.3%)
Cerebrocortical/ perivascular oedema with astrocytosis	17, 23, 30, 35, 37, 38, 13, 16	8/45 (17.8%)
Neuronal lipofuscinosis.	11, 12, 13, 14, 24, 27, 45	7/45 (15.6%)
Perivascular ceroid/hemosiderin pigment	24, 41, 45	3/ 45 (6.7%)
Satellitosis	12, 17, 45	3/ 45 (6.7%)
Neuronal necrosis	26, 32, 42	3/ 45 (6.7%)
Lymphoplasmacytic meningoencephalitis	31, 37	2/45 (2.3%)
Gliosis	41, 44	2/45 (2.3%)
Spongiosis	27	1/45 (2.2%)
Intramyelinic oedema with axonal degeneration	17	1/45 (2.2%)
Multifocal intravascular nematode eggs	16	1/45 (2.2%)
Spheroids	12	1/45 (2.2%)
- *Spinal cord*		
Meningeal congestion with hemorrhage	39, 41	2/45 (2.3%)
Vacuolization of the neuroparenchyma	41	1/45 (2.2%)
Focal perineural gliosis	34	1/45 (2.2%)
- *Myelenencephalon*		
Vacuolization of the neuroparenchyma	41	1/45 (2.2%)

Table S4. Gross and histologic pathologic findings in cardiac tissue from 45 kogiid whales (*Kogia breviceps, K. sima*) stranded din the Canary Islands (1999-2018).

	Gross Findings						Histologic Findings				
Animal No.	Cardiomegaly	Congestion/Hemorrhage	Hemopericardium	Hydropericardium	Fibrosis	Hypertrophy	Degeneration/Necrosis	Juxtanuclear Vacuolization	Intercellular Edema	Neutrophilic Myocarditis	PDA
1	Yes	No	No	No	No	No	No	No	No	No	No
2	Yes	No	No	No	No	No	No	No	No	No	No
5	Yes	Yes	No	No	Yes	No	No	No	No	No	No
11	Yes	No	No	No	No	Yes	No	No	No	No	No
12	No	Yes	No	No	No	Yes	Yes	No	No	No	No
13	No	No	No	Yes	No	No	Yes	No	No	No	No
14	No	Yes	No	No	No	Yes	No	Yes	No	No	No
16	Yes	No	No	No	No	Yes	Yes	Yes	Yes	No	No
17	No	Yes	No	No	No	No	Yes	No	Yes	No	No
19	No	No	No	Yes	No	Yes	Yes	No	No	No	No
20	Yes	No	No	No	No	Yes	Yes	No	No	No	No
22	No	Yes	No	Yes	No	No	Yes	No	No	No	No
23	Yes	No	No	No	No	No	Yes	No	Yes	No	No
24	No	No	No	No	Yes	No	Yes	No	No	No	No
25	Yes	No	No	Yes	No	No	Yes	Yes	No	No	No
26	No	No	No	No	No	No	Yes	No	No	No	Yes
27	No	No	No	Yes	No	No	Yes	No	No	No	No
28	Yes	No	Yes	No	Yes	No	No	Yes	No	No	No
30	No	Yes	No	No	No	No	Yes	No	Yes	No	No

31	Yes	Yes	No	No	No	No	No	No	Yes	No	No
32	Yes	No	No	No	No	No	No	No	No	No	No
33	Yes	No	No	No	No	No	No	No	No	No	No
35	Yes	No	No	No	No	No	Yes	No	No	No	No
36	No	No	Yes	No	No	No	No	No	No	No	No
37	No	Yes	No	No	No	No	Yes	Yes	No	No	No
38	No	Yes	No	No	No	No	No	No	No	No	No
39	No	Yes	No	No	Yes	No	No	No	No	No	No
40	No	No	No	No	No	No	Yes	No	No	No	No
41	No	Yes	No	Yes	No	No	Yes	No	No	No	Yes
42	No	No	No	No	Yes	Yes	Yes	Yes	No	Yes	No
43	No	No	No	No	Yes	No	No	No	No	No	No
44	No	No	No	No	No	No	Yes	No	No	No	No
45	Yes	Yes	No	No	No	No	Yes	No	No	No	No
Total	14 / 45	12 / 45	2 / 45	6 / 45	6 / 45	7 / 45	20 / 45	6 / 45	5 / 45	1 / 45	2 / 45
